# Incomplete estimates of genetic diversity within species: Implications for DNA barcoding

**DOI:** 10.1002/ece3.4757

**Published:** 2019-02-16

**Authors:** Jarrett D. Phillips, Daniel J. Gillis, Robert H. Hanner

**Affiliations:** ^1^ School of Computer Science University of Guelph Guelph Ontario Canada; ^2^ Centre for Biodiversity Genomics Biodiversity Institute of Ontario University of Guelph Guelph Ontario Canada; ^3^ Department of Integrative Biology University of Guelph Guelph Ontario Canada

**Keywords:** cytochrome *c* oxidase subunit I, DNA barcoding, sampling, species, statistics, sufficiency

## Abstract

DNA barcoding has greatly accelerated the pace of specimen identification to the species level, as well as species delineation. Whereas the application of DNA barcoding to the matching of unknown specimens to known species is straightforward, its use for species delimitation is more controversial, as species discovery hinges critically on present levels of haplotype diversity, as well as patterning of standing genetic variation that exists within and between species. Typical sample sizes for molecular biodiversity assessment using DNA barcodes range from 5 to 10 individuals per species. However, required levels that are necessary to fully gauge haplotype variation at the species level are presumed to be strongly taxon‐specific. Importantly, little attention has been paid to determining appropriate specimen sample sizes that are necessary to reveal the majority of intraspecific haplotype variation within any one species. In this paper, we present a brief outline of the current literature and methods on intraspecific sample size estimation for the assessment of COI DNA barcode haplotype sampling completeness. The importance of adequate sample sizes for studies of molecular biodiversity is stressed, with application to a variety of metazoan taxa, through reviewing foundational statistical and population genetic models, with specific application to ray‐finned fishes (Chordata: Actinopterygii). Finally, promising avenues for further research in this area are highlighted.

## 1 INTRODUCTION

One of the most fundamental problems underpinning much of modern molecular biodiversity research is the issue of determining optimal levels of sampling effort that are required in order to adequately characterize biological sequence variation at the species level. Molecular genetic studies of biodiversity that utilize mitochondrial DNA (mtDNA) marker variation for the purpose of characterizing existing species genetic diversity are particularly sensitive to sample sizes. Four fundamental evolutionary forces act to alter the genetic composition of species populations: migration/gene flow, mutation, natural selection and random genetic drift. The effect of genetic drift on species populations is most evident when population sizes are small, as in the case of a recent bottleneck or founder event, resulting in the rapid loss of genetic diversity. Species differ both in their evolutionary histories and in their geographic distributions; therefore, the question of accurately determining how many samples to include in order to observe a wide range of species genetic variation has been an ongoing area of interest and research. This is an important question deserving of more attention. Accurate determination of within‐species (intraspecific) sample sizes for mtDNA diversity estimation permits detailed analyses to be undertaken at the phylogenetic and phylogeographic levels in order to infer key biological processes such as isolation, dispersal and speciation (Avise et al., 1987; Dixon, 2006; Funk & Omland, 2003). Aside from addressing purely biological questions, the issue of determining optimal sampling strategies and sample sizes for genetic variation assessment at the species level also manifests at applied socioeconomic scales, particularly in the detection of food or natural health product fraud and in the monitoring of aquatic and terrestrial ecosystems (Hunter et al., 2015).

Within the field of biodiversity science, researchers have long recognized the importance of sampling design in order to achieve a study's objectives. According to Lindblom (2009), well‐developed sampling designs within the field of molecular biodiversity science should be formulated around three basic areas: research study questions, research study aims and taxonomic focus. In addition to these three areas, Costa, Corneleo, and Stefenon (2015) point to further considerations: planning the number and geographic distribution of specimens to be sampled, the category and number of genetic loci to be examined, and the spatial distribution and number of individuals to be sampled within each species’ population. While there is a lack of clear sampling guidelines currently in place for optimal spatio‐temporal assessment of species populations, Pante et al. (2015) argue that such schemes should be guided by adequate coverage of both the putative geographic/ecologic range of the species under study, and potentially closely related species over its entire range. Given that much of species spatio‐temporal metadata is not reported alongside genetic data, such assessments become problematic unless community standards and practices are improved (Hanner, 2005; Naaum et al., 2015; Strohm, Gwiazdowski, & Hanner, 2016). Where this becomes particularly important is in the development and design of species‐specific real‐time polymerase chain reaction (qPCR) primers and probes, for integration within environmental DNA (eDNA) assays for instance. This is especially the case if such tools are to be continuously implemented within regulatory or forensic settings such as the Canadian Food Inspection Agency (CFIA) (Shehata, Naaum, Garduno, & Hanner, 2018) and the US Food and Drug Administration (USFDA), as the success of such methods depends greatly on the extent of geographic coverage of species genetic diversity.

The overall goal of sampling is to make inferences concerning a population of interest based only on information contained within finite samples drawn from the larger population. This is done though estimating population parameters such as the population mean (*μ*) using the sample mean ($\overset{¯}{x}$). One example, relevant to molecular population genetics, is the calculation of average pairwise distances based on Nei's estimator of nucleotide diversity (*π*) (Nei & Li, 1979). Under the Frequentist statistical paradigm, the minimum sample size that is required to estimate a population mean, from a Normal distribution, is given by Adcock (1997)

(1) $$n\geq {\left(\frac{z_{\alpha /2}\sigma }{d}\right)}^{2}$$

where $z_{\alpha /2}$ is the appropriate critical value to estimate *μ* with a level of significance of 1−*α*, $\sigma^{2}$ is the population variance and *d* is the desired margin of error. From the above equation, the required minimum sample size is controlled by the experimenter through the margin of error. A smaller margin of error results in a larger value of *n*. Similarly, predicting *n* with a higher level of accuracy can be achieved through narrowing *d*. Sample sizes that are computed from the above equation serve as a baseline requirement prior to conducting any quantitative study of interest. Depending on the sampling scheme, for instance stratified sampling, other formulas exist for the appropriate calculation of necessary sample sizes.

In determining the most appropriate sample size required for a particular study, a crude rule of thumb that is often used in statistics and other scientific disciplines pertains to the use of a sample size of at least *n* = 30 when making comparisons among study groups or when deciding to use probabilities derived from the Standard Normal distribution (Cohen, 1990). Unfortunately, adequate sample sizes, while widely viewed as being central to a given biodiversity research study, are often neglected in practice (Lenth, 2001). In such cases, this may be due to, for example, costs associated with or resources required for adequate specimen collection (Cameron, Rubinoff, & Will, 2006; Hortal & Lobo, 2005; Muirhead et al., 2008).

Statistical power analysis can be employed to help shed light on sample sizes required in order to detect a given effect prior to carrying out a scientific study. Power, which is defined as the complement of the type II error rate (*β*), depends on four factors: effect size (ES), significance level/type I error rate (*α*), sample size (*n*) and population standard deviation (*σ*) through the proportionality (Di Stefano, 2003)

(2) $$\left(1-\beta \right)\propto \frac{\text{ES}\times \alpha \times \sqrt{n}}{\sigma }.$$

Effect size is the difference between an observed quantity and one hypothesized under a null distribution. Larger deviations lead to greater power to detect real effects. It is easily seen from the above proportionality that larger values of effect size, significance level and sample size all generate higher levels of statistical power, whereas increasing population standard deviation results in loss of power. Together with the sample size equation discussed previously (Equation (1)), many factors are at play in determining the most appropriate sample size needed for a given study.

Any sampling scheme that is carried out will be subject to systematic error. Sampling (ascertainment) bias is an important factor to consider in this regard because it can lead to under‐ or overestimation of population parameters. Ascertainment bias describes the tendency of certain individuals to be less likely sampled than others (Parr, Guralnick, Cellinese, & Page, 2012) and is common in molecular biodiversity studies (Hanner, Becker, Ivanova, & Steinke, 2011; Muirhead et al., 2008; Mutanen et al., 2016; Wilkinson et al., 2017). This can occur, for example, when sampling is restricted to certain geographic regions (Muirhead et al., 2008) or to particular species (e.g., those known to be of conservation importance) (Hanner et al., 2011). Sampling bias can be minimized through increasing the geographic breadth of a study, in addition to targeting representative taxa with large specimen sample sizes.

The present review briefly examines current approaches for species genetic variation assessment as it relates to the estimation of intraspecific sample sizes for DNA barcoding. Specifically, the focus will be on COI DNA barcode haplotype sampling completeness. Few studies have focused on DNA barcode sample size prediction for wide‐ranging taxa in this regard. Here, methods of haplotype variation assessment are first covered. This is then followed by an examination of existing studies, with particular consideration of important findings to date within the literature. Finally, promising new avenues for further research are explored.

## 2 CURRENT METHODS

### 2.1 Methods to assess haplotype variation

#### 2.1.1 Haplotype diversity

Genetic diversity is manifested within species in several ways. One way is through haplotype variation. While there are many different definitions of what constitutes a haplotype, in the broadest sense, a haplotype is a unique DNA sequence that differs from others at one or more basepair positions within and between species. Nei's (1987) haplotype diversity (*h*), which is a widely used approach to measuring genetic variation within species populations, is given by the equation

(3) $$h=\frac{n}{n-1}\left(1-\sum_{i}p_{i}^{2}\right).$$

where *p*_*i*_ is the frequency of the *i*th haplotype in the sample. Two interpretations of *h* are that it expresses the probability of observing a previously unseen haplotype upon sampling a new individual (Wares & Pappalardo, 2015) or that it represents the probability that two haplotypes, selected at random from a sample of *n* DNA sequences, are distinct (Goodall‐Copestake, Tarling, & Murphy, 2012). Haplotype diversity can also be quantified using the absolute number of haplotypes (*H*). Both *h* and *H* are greatly affected by levels of sampling intensity within species. In particular, undersampling can cause these measures to become under‐ or overestimated (Goodall‐Copestake et al., 2012). Several other approaches are in wide use to aid researchers in assessing levels of standing genetic variation existing within species populations. Two of these are haplotype networks and haplotype accumulation curves.

#### 2.1.2 Haplotype networks

A widely used approach to assessing levels of genetic variation within and between species is through the construction of haplotype networks (Templeton, Crandall, & Sing, 1992). Haplotype networks accurately represent differences existing among sampled haplotypes through grouping identical DNA sequences within the same vertex. The size of a given vertex is proportional to the number of DNA sequences it contains. Divergent haplotypes are connected via edges that display the number of mutational differences separating adjacent vertices.

**Figure 1:**
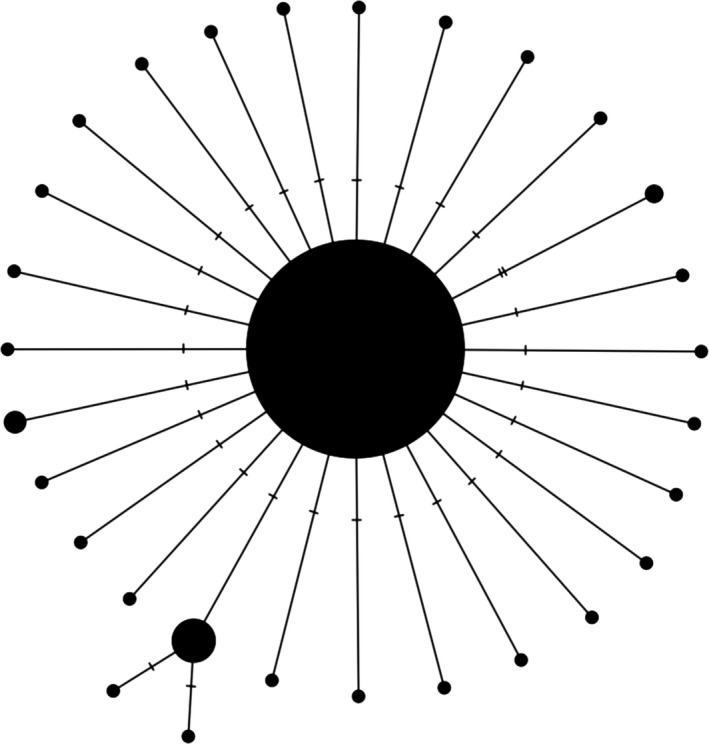
Longfin damselfish (*Stegastes diencaeus*) TCS (Templeton et al., 1992) haplotype network depicting an overall skewed distribution of observed haplotypes. Sizes of circles reflect the number of DNA sequences contained within each vertex. Tick marks indicate the number of mutational differences separating sampled haplotypes. DNA barcode sequence data used in the generation of the network were taken from supplemental material accompanying Phillips et al. (2015). The software PopArt (Leigh & Bryant, 2015) was used to create the haplotype network

Haplotype networks are appealing because they can be used to infer potential cryptic diversity within a taxon or interspecific hybridization between allopatric (i.e., reproductively isolated) species, but interpretation can sometimes become difficult when multiple species cluster together into one or multiple nodes or subnetworks (Hanner, Floyd, Bernard, Collette, & Shivji, 2011; Hart & Sunday, 2007; Wong, Shivji, & Hanner, 2009) or when ambiguous/missing nucleotide data are present within DNA sequences (e.g., Ns or gaps (–)) (Joly, Stevens, & van Vuuren, 2007). While haplotype networks, such as the one shown in Figure 1, cannot give a direct indication of the level of sampling completeness for a given species, the presence of numerous rare haplotypes suggests gross undersampling of intraspecific genetic variation (or alternatively PCR/sequencing error).

#### 2.1.3 Haplotype accumulation curves

Assessing the completeness of intraspecific haplotype sampling can be carried out through generating haplotype accumulation curves. Such curves are analogous to rarefaction curves used in studies of species richness (Gotelli & Colwell, 2001) and depict the degree of asymptotic behavior as a function of both the number of specimens sampled and the cumulative mean number of haplotypes accumulated. Initially, accumulation curves will increase very rapidly since many new haplotypes will be captured for a given species with minimal sampling effort, but haplotype recovery slows drastically as sampling depth is increased because many haplotypes that are found will have already been observed previously. Thus, species curves showing rapid saturation strongly suggest that the majority of haplotype diversity has been uncovered, whereas those curves displaying little to no evidence of reaching an asymptote indicate that further sampling is required (Zhang, He, Crozier, Muster, & Zhu, 2010). Deciding whether a species should be further sampled can be deduced from the magnitude of the slopes calculated using a fixed number of points occurring on the end of the curve (e.g., 10 in the case of Phillips, Gwiazdowski, Ashlock, & Hanner, 2015; Young, Behan‐Pelletier, & Hebert, 2012). Slopes near or below a predefined threshold, for example, 0.01 (i.e., equivalent to observing one new haplotype for every 100 DNA sequences), suggest that additional sampling is unlikely to reveal any new haplotypes, whereas those species curves with slopes above 0.1 (i.e., observing one new haplotype for every 10 DNA sequences), strongly indicate that further sampling is necessary (Hortal & Lobo, 2005).

One obvious problem that arises in the use of haplotype accumulation curves to gauge species genetic diversity and levels of sampling effort, however, is the fact that the functional form of such curves is not known and can differ widely across taxa (Phillips et al., 2015). Furthermore, deciding on appropriate curve slope thresholds necessary for adequate sampling coverage is largely arbitrary (Hortal & Lobo, 2005). While various parametric model curve‐fitting approaches, such as the power, negative exponential, and Michaelis–Menten functions, have been heavily employed and debated in the literature to model species–area relationships (Dengler, 2009; Tjørve, 2003) or species richness, no single approach yet exists that can be readily applied to determine sample sizes that are likely required for intraspecific genetic variation assessment.

A second, lesser‐investigated issue, relates to the fact that haplotype accumulation curves are not spatially explicit. Thus, it becomes difficult to account for correlations that may exist at the subpopulation or higher taxonomic levels. This has been noted in past studies of species richness employing species accumulation and rarefaction curves (Bevilacqua, Ugland, Plicanti, Scuderi, & Terlizzi, 2017; Chiarucci, Bacaro, Ricotta, Palmer, & Scheiner, 2009; Terlizzi, Anderson, Bevilacqua, & Ugland, 2014).

### 2.2 Sampling models for genetic diversity prediction

In addition to qualitative approaches to assessing standing genetic variation within species, a number of quantitative models to estimate required sample sizes for overall genetic diversity assessment have been proposed. These include Frequentist, Bayesian, and coalescent models.

Holt, Stoneberg Holt, and Bureš (2007) reviewed several Frequentist and Bayesian statistical methods of sample size determination for intraspecific haplotype diversity assessment that are most informative over large geographic ranges. The authors note that a lower bound on the probability of sampling a dominant haplotype in a sample of size *n* with significance level *α* is given by the inequality

(4) $$p\geq \sqrt[{n}]{\alpha }$$

Grewe et al. (1993) employed an equivalent approach to Holt et al.'s (2007) study through utilizing a binomial sampling model to determine the minimum sample size required to assess mtDNA variation in Lake Ontario lake trout (*Salvelinus namaycush*) stocks according to the equation

(5) $$n=\frac{\ln(1-\beta )}{\ln(1-p)}$$

where *p* is the frequency of a given haplotype, and *β* is the desired confidence level. The authors found that *n* = 60 individuals are likely needed to be randomly sampled in order to observe a single haplotype having a frequency of at least *p* = 5% with *β* = 95% confidence. It is worth noting that this figure increases to *c*. 460 individuals for a haplotype occurring at frequency of 1% with 99% confidence (Grewe et al., 1993). This marked increase in sample size is not surprising given that one would need to sample many more individuals in order to be certain that the majority of rare haplotypes have been uncovered. It is important to note, however, that Grewe et al. (1993) sampled individuals from six different but highly divergent trout strains, each displaying high degrees of population substructure. Population subdivision likely will have an effect on the estimation of required sample sizes needed to gauge levels of standing genetic variation at the species level.

Similar magnitudes of sample sizes were found by Austerlitz et al. (2009), who employed coalescent theory (Kingman, 1982), in order to determine the probability of adequately sampling all genetic variation of a species with sample size *n*. Coalescent theory attempts to trace the lineage of an ancestral allele (termed the Most Recent Common Ancestor, MRCA) backwards in time within a gene genealogy. Under a geometric distribution, this probability is given by the equation (Austerlitz et al., 2009)

(6) $$p=\frac{n-1}{n+1}.$$

From the above equation, only *n* = 39 individuals are required to be sampled at random in order to observe *p* = 95% of all genetic diversity for a species. It should be noted however that even with increasing sample sizes, one's confidence in having sampled all of a species’ genetic diversity approaches closely, but never actually reaches, 100% (Austerlitz et al., 2009). This is illustrated by the finding that the required sample size increases to *n* = 1999 individuals necessary to observe *p* = 99.9% of the total genetic diversity that exists for a given species using Equation (6). This can be explained by the fact that individual haplotypes for a given species become much more difficult to recover as the intensity of specimen sampling is increased because intraspecific genetic variation is expected to increase as a result. The coalescent, as a large‐scale sampling model, has found wide application in DNA‐based approaches to species identification and delimitation, most notably DNA barcoding (Hubert & Hanner, 2015).

### 2.3 DNA barcoding

Since its conception in 2003, DNA barcoding (Hebert, Cywinska, Ball, & de Waard, 2003) has risen to become the largest taxonomically driven biodiversity initiative to date aimed at identifying and cataloging all assemblages of multicellular life on the planet. DNA barcoding is a genomic technique that relies on DNA sequence variation within short, standardized gene regions in order to rapidly identify specimens to the level of species and to discover new species. The ideal DNA barcode is one that is found in all organisms, readily distinguishes between taxa, and is easily amplified, sequenced, and aligned. In animals, the agreed‐upon marker of choice for taxon assignment is a *c*. 650 basepair (bp) fragment from the 5′ end of the mitochondrially encoded cytochrome *c* oxidase subunit I (COI) gene. Mitochondrial loci like COI are particularly suitable as genetic markers for DNA barcoding because they are fast evolving, highly conserved across taxa, present in high copy number, haploid, maternally inherited, lack introns, display few insertion—deletion (indel) mutations, and experience little to no gene recombination (Hebert, Ratnasingham, & de Waard, 2003; Hebert et al., 2003).

The primary goal of DNA barcoding has been to develop a publicly accessible species reference sequence library to aid in the identification of unknown specimens and accelerate the discovery of potentially undescribed taxa. Obtaining adequate sample sizes for building accurate and reliable specimen reference libraries has culminated in the development of the Barcode of Life Data Systems (BOLD; http://www.boldsystems.org) (Ratnasingham & Hebert, 2007) as the largest collection of user‐curated species sequence data specifically for DNA barcoding currently available on the World Wide Web. At present (as of 1 May 2018), BOLD holds over six million DNA barcode records from over 250,000 named species. Certain taxa are well represented in BOLD with upwards of hundreds of barcode sequences for some species. Despite this, barcode reference libraries within BOLD remain largely incomplete, even for the most well‐sampled taxa such as fishes and insects. As such, comprehensive coverage of species genetic diversity is still decades away (Wilkinson et al., 2017). Wilkinson et al. (2017) points to strong ascertainment bias as the most likely explanation for this. In the early days of BOLD, DNA barcode sequence acquisition was high, due to the fact that over 75% of taxon records were mined from already well‐established sequence databases such as GenBank (Wilkinson et al., 2017).

### 2.4 The importance of sampling to DNA barcoding

DNA barcoding works in practice because interspecific (between species) variation is usually much greater than intraspecific (within‐species) divergence (Meyer & Paulay, 2005; Stoeckle & Thaler, 2014). While this observed “barcoding gap” (Meyer & Paulay, 2005) is a necessary criterion for successful taxonomic resolution using distance‐based methods, it may not be a sufficient one for other molecular approaches (e.g., those employing tree‐ or character‐based techniques). Cases are well documented where considerable overlap/separation between (maximum) intraspecific variation and (minimum) interspecific divergence exists (Hebert, Stoeckle, Zemlak, & Francis, 2004; Hubert & Hanner, 2015). Undersampling can greatly exaggerate the existence of the barcode gap. The inclusion of small sample sizes over large geographic ranges has the effect of obscuring existing mitochondrial sequence diversity at the species level since the finding of divergent haplotypes may be the result of poorly sampled panmictic (i.e., randomly mating) intraspecific variation (Clare, Lim, Fenton, & Hebert, 2011). Compared to regional scales, with increasing sampling effort across wider spatial scales, intraspecific variation is expected to increase, whereas interspecific divergence will decrease in effect since more closely related species will tend to be found due to allopatric speciation being a dominant mode of diversification (Bergsten et al., 2012; Pentinsaari, Hebert, & Mutanen, 2014).

How much variation is actually needed to separate species is not known with certainty because intraspecific sampling has generally been limited to narrow geographic locales. Hebert et al. (2003) proposed that barcode sequences exhibiting at least 2% nucleotide divergence should be designated as being from distinct species. Intraspecific distances larger than 2% suggest the presence of cryptic species, whereas those smaller than 2% is evidence for evolutionarily young species with a recent origin (i.e., retention of ancestral polymorphisms due to incomplete lineage sorting), hybridization/introgression or inadequate taxonomy (e.g., cryptic species or species synonymy) (Hubert & Hanner, 2015). In BOLD, query sequences are matched to reference barcodes based on a genetic distance heuristic of 1% (Ratnasingham & Hebert, 2007). The use of such threshold estimates for species separation is arbitrary and is often applied to a wide variety of taxa, regardless of species life histories. A later estimate of ten times the mean intraspecific distance (the so‐called “10× rule”) was given by Hebert et al. (2004). Unlike the previously suggested estimate of 2% sequence divergence, the 10× rule makes use of all available taxon sequences within a dataset in order to calculate an appropriate limit for species separation. Despite this, the 10× rule has been met with criticism: Collins and Cruickshank (2013) suggest consideration of the maximum intraspecific distance and the minimum interspecific divergence (i.e., nearest neighbor distance) for each species under investigation. The use of lower thresholds for species discovery may falsely inflate existing genetic diversity, whereas the adoption of higher cutoffs would likely be too conservative for reliable detection of cryptic species (April, Mayden, Hanner, & Bernatchez, 2011). It is well understood however that the most appropriate cutoff necessary to accurately diagnose species on the basis of sequence variation is strongly taxon‐dependent (Hebert et al., 2003; Hickerson, Meyer, & Moritz, 2006; Meyer & Paulay, 2005) and will become more precise with increased sampling effort.

DNA barcoding has its roots in the historic disciplines of Darwinian evolutionary theory, population genetics, and phylogenetics: The coalescent is a modern interpretation that reconciles these domains (Rosenberg & Nordborg, 2002). While genetic distance‐based approaches to species delimitation are commonplace within barcoding studies because they scale well to large taxon datasets, early‐proposed arbitrary separation methods like the 2% or 10× rule completely ignore evolutionary relationships that exist among closely related species. Objective tools for the delimitation of species are well known and generally fall into three overlapping categories: phylogenetic, coalescent, and phylogenetic‐coalescent (Hubert & Hanner, 2015). The well‐known neighbor‐joining clustering method was advocated for in the early barcoding literature as a means of confirming the presence of reciprocal monophyly across sampled taxa. More recently, novel bioinformatic algorithms, most notably distance‐based approaches such as Automatic Barcode Gap Discovery (ABGD; Puillandre, Lambert, & Brouillet, 2011) and tree‐based methods including variants of the Generalized Mixed Yule Coalescent (GMYC; Monaghan et al., 2009; Pons et al., 2006), have been put forth in order to facilitate species separation, an otherwise daunting task for even the most highly skilled and knowledgeable taxonomist. ABGD is a nonparametric technique of partitioning species on the basis of the barcode gap using DNA sequences. On the other hand, GMYC is a likelihood‐based method that relies on the premise that bifurcation (i.e., fully resolved branching) within ultrametric species trees is indicative of speciation/diversification events, and therefore suggests the presence of undescribed taxa. A key factor in the success of such methods is sample size, and few groups have been so extensively inventoried (Hubert & Hanner, 2015). For example, GMYC is especially prone to the under‐ or overestimation of putative species, which can be magnified due to differences in effective population sizes as well as historical versus contemporaneous patterns of migration/gene flow among subpopulations (Lohse, 2009; Papadopoulou, Monaghan, Barraclough, & Vogler, 2009). Thus, sufficient sampling is paramount. Often, researchers would like to know whether all unique haplotypes within a lineage or deme have been adequately sampled; unfortunately, this is complicated by the fact that the majority of species are both geographically widespread and rare. As a result, given that ascertainment and operational biases are inevitable (Mutanen et al., 2016), an extensive sampling of all local populations that comprise a given species is unrealistic, even under the best situations (e.g., strong research budget, easy access to sampling locations). Thus, whenever possible, a more comprehensive sampling of study sites is required in order to avoid false positives/negatives and to reveal divergent haplotypes that may have been missed with spatially narrower sampling routines (Monaghan et al., 2009). Incorporation of coalescent and population genetics theory can aid in informing researchers on broad macro‐level processes that may be at play in shaping trends seen within haplotype accumulation curves on the basis of extant patterns of intraspecific genetic diversity.

The Barcode Index Number framework for animals, first introduced by Ratnasingham and Hebert (2013), represents a potentially novel approach to addressing the issue of sample sizes necessary for barcoding initiatives. The BIN system partitions COI barcodes into distinct Operational Taxonomic Units (OTUs) on the basis of the REfined Single Linkage (RESL) clustering algorithm and Markov clustering (Ratnasingham & Hebert, 2013). BINs comprise high‐quality sequences linked to BARCODE compliant records. The BARCODE standard currently in place stipulates that only barcode sequences with read lengths of at least 500 bp and containing less than 1% ambiguous nucleotides are designated unique BIN clusters (Hanner, 2005). While BINs generally show high concordance with actual biological species, they can be further employed to gauge instances of suspected cryptic species diversity, especially in the cases where intraspecific distances are not clear‐cut. Species that fall into two separate BINs (termed a SPLIT) is evidence that they are being overlumped. Further, the occurrence of rare BINs (i.e., those represented by a single specimen) may be the result of limited sampling (Hausmann et al., 2013; Huemer, Mutanen, Sefc, & Hebert, 2014). Stand‐alone BINs may also reflect sequencing errors in the form of very low‐frequency (VLF) variants or cryptic pseudogenes (Stoeckle & Kerr, 2012; Stoeckle & Thaler, 2014). Increased sampling coverage can be beneficial in such instances, as true biological variation is less likely to be misidentified as artificial biological variation and unintentionally flagged as potential VLFs.

### 2.5 Consideration of species’ life histories

Life history traits, particularly those pertaining to reproductive strategies and sex determination, in well‐studied metazoan taxa such as fishes, insects, and herpetofauna, are presumed to play a significant role in observed patterns of mtDNA barcode sequence variation at the species level. For instance, the high occurrence of haplodiploidy, a mode of inheritance whereby females develop from fertilized eggs (hence are diploid), while males arise from unfertilized eggs (therefore are haploid), is common across many insect orders such as Hymenoptera, and may explain the large abundances and varying (effective) population sizes seen in representative species that ultimately drives speciation and hybridization (Hebert, Ratnasingham, & Zakharov, 2016). Similar “exceptions to the rule,” such as (asexual) modes of parthenogenesis (e.g., unfertilized eggs producing female‐only offspring in Squamata such as species of whiptail lizards), or paternal/biparental organelle inheritance in bivalve molluscs (e.g., mussels of the genus *Mytilus*), will likely help inform researchers on the required level of sampling depth needed to fully characterize broad ranges of COI haplotype diversity in taxa that do not otherwise conform to traditional mtDNA inheritance patterning (i.e., strictly maternal lineage), and thus prevent the naïve implementation of recommendations of any one statistical approach employed in the calculation of intraspecific sample sizes for accurate specimen assignment and rapid species delineation. As an example, because parthenogenetic species display lower standing genetic diversity compared to fully sexually reproducing species (as a result of being exact clones of their parent due to lack of chromosomal recombination) (Bengtsson, 2003), haplotype frequencies aside, the observation of the faster approach of haplotype accumulation curves to an asymptote is expected. Thus, species exhibiting such mechanisms will require reduced levels of sampling effort. Such a result can be invoked through consideration of Muller's ratchet, as the irreparable accumulation of deleterious mutations that are fixed by genetic drift within asexual genomes directly limits the ability of a species to survive and reproduce (Felenstein, 1974; Muller, 1964).

## 3 Key findings

### 3.1 DNA barcoding and sample size: past studies

The ability of DNA barcodes to uncover levels of standing genetic variation within species is strongly influenced by the scale of specimen sampling, which has been recognized as a major barrier to the success of DNA barcoding since its early days (Hebert et al., 2004; Meyer & Paulay, 2005; Ward, Zemlak, Innes, Last, & Hebert, 2005). In spite of this, global barcoding efforts have only been partially successful in capturing the full extent of COI barcode variation in animals due to the majority of studies forgoing deep taxon sampling in favor of maximizing the number of different taxa sampled (Matz & Nielsen, 2005; Zhang et al., 2010). Sample sizes of a few individuals per species (typically in the range of 5–10, but one or two specimens is not uncommon since these are often the only representatives available, either due to unclear species boundaries or limited geographic sampling of intraspecific variation) are widespread in barcoding studies (Hajibabaei, Singer, Hebert, & Hickey, 2007; Matz & Nielsen, 2005; Zhang et al., 2010). Recommended sample sizes currently in place are by no means sufficient since species abundance is often skewed geographically/ecologically. For example, five specimens per species per FAO (Food and Agriculture Organization) region were initially suggested by the Fish Barcode of Life (FISHBOL; Ward, Hanner, & Hebert, 2009) initiative, but the sampling of up to 25 individuals or more may be necessary for some species exhibiting widespread distribution patterns (Becker, Hanner, & Steinke, 2011; Steinke & Hanner, 2011). Similarly, in assessing haplotype and nucleotide COI variation across wide‐ranging animal taxa, Goodall‐Copestake et al. (2012) note that a sample size of five individuals per species population was adequate to differentiate between extremes of *h*, but as many as 25 specimens would need to be collected in order to achieve maximum accuracy. Jin, He, and Zhang (2012), and Matz and Nielsen (2005) both point to a sample size of 12 specimens, whereas Ross, Murugan, and Li (2008) suggest that sampling five or more reference barcodes is sufficient for accurate species identification. Bias toward low sample sizes observed for most species may be the result of many factors (see Bucklin, Steinke, and Blanco‐Bercial (2011) for a concise summary in marine metazoa), including the presence of cryptic diversity, amplification of nonfunctional gene copies (i.e., pseudogenes/nuclear–mitochondrial inserts (NUMTs)), contamination by foreign DNA from other species (e.g., bacterial symbionts such as *Wolbachia*), insertion–deletion (indel) mutations, or errors arising from PCR/sequencing runs (Goodall‐Copestake et al., 2012). Molecular diagnosis of specimens to the species level using DNA barcoding is not definitive; numerous technical sources of error exist that can hamper the ability of reliable taxon assignment, in particular, misidentifications, sequencing errors, and lack of taxonomic metadata (e.g., inclusion of GPS coordinates, record linkage to a voucher specimen). While such factors are likely to occur infrequently for interspecific barcodes, this is not the case for intraspecific datasets. Taken together, biases in sample sizes will likely be considerable. In certain cases, the occurrence of biological phenomena can lead to problems encountered later on in the laboratory, specifically during the sequence amplification stage using PCR. A well‐known example of this is the symbiotic association of the bacterium *Wolbachia* with insects. Integration of *Wolbachia* within host genomes of various Hymenoptera, Diptera, and Lepidoptera can cause fluctuations in intraspecific distances (Smith et al., 2012) and thus observed haplotype diversity between infected and uninfected hosts (Chen et al., 1984). Misamplification of host sequences for bacterial symbionts is widely encountered, as is the amplification of pseudogenes/NUMTs. Technical sources of error such as expert taxonomic misidentifications, sequence contamination, and errors arising from the amplification/sequencing process can be controlled and can be minimized to a degree. Two critical steps in avoiding such issues are as follows: (a) the construction of an NJ tree in order to pinpoint potentially misidentified specimens and/or sequence contaminants (as opposed to solely being used in the establishment of reciprocal monophyly, as argued by Collins and Cruickshank (2013)) and (b) the careful inspection of BOLD specimen trace files in order to resolve noisy sequence regions that inflate estimates of standing genetic variation through the introduction of functional (heteroplasmic) sequence variation (as in e.g., Hebert, Penton, Burns, Janzen, & Hallwachs, 2004) and/or nonexistent low‐frequency species haplotypes occurring in high abundance (Stoeckle & Kerr, 2012). The effect of these on generated haplotype accumulation curves is delayed saturation to an asymptote due to larger required sample sizes. Combined with initially large numbers of specimens within intraspecific datasets (e.g., *N* > 100), this effect can be quite substantial. As BOLD is ever‐evolving, in part due to the sheer volume of DNA barcode sequences being added on a daily basis, it is crucial that suspected errors within taxon records be dealt with in a timely manner (e.g., through community users flagging problematic records for closer examination by submitters), so that sequence integrity is not compromised. While the issue of determining adequate sample sizes for molecular species diagnosis has largely been aimed at animal taxa, Liu, Provan, Gao, and Li (2012) explored optimal sample sizes needed for plant DNA barcoding. It was found that relatively small sample sizes were adequate to recover sequence variation in slowly evolving genes (two or three sequences per species population for matK), whereas higher numbers are necessary for rapidly evolving markers (minimum of 10, 8, and 6 individuals per population for trnH‐psbA, trnL‐trnF, and ITS, respectively) (Liu et al., 2012). Further, the authors found that a sample size of 8–10 individuals per species across the entire geographic range appears sufficient for *Taxus* barcoding. Unfortunately, such small sample sizes, likely the result of low information content due to the high presence of sequence artifacts (e.g., indels within mitochondrial/plastid markers), often lack discriminatory power that is needed for accurate identification of specimens on the basis of genetic polymorphism with DNA barcodes.

To date, few studies explicitly exploring simulated sample sizes for DNA barcoding in wide‐ranging animal taxa have been conducted. One of the first studies to examine the issue of sample sizes for DNA barcoding via haplotype accumulation curves was conducted by Zhang et al. (2010) using a modified form of the Michaelis—Menten equation. Using this method, the authors found that the random sampling of 250–1,188 individuals from the Costa Rican skipper butterfly (*Astraptes fulgerator*) cryptic species complex are likely needed in order to detect 95% of all genetic diversity for this species based on an initial sample size of 407 individuals. Conversely, the same authors found that 156–1,985 specimens were needed to retrieve 95% of COI variation using simulated island (Wright, 1951) and stepping‐stone (Kimura & Weiss, 1964) coalescent models across three distinct subpopulations and under varying effective population sizes. In addition, a sample size outlier of only 47 individuals was found for one subpopulation of *A. fulgerator* butterflies. The authors note that this may be due to the low level of genetic variation observed in this population: Only two haplotypes were observed across 14 sampled individuals. In contrast, a later study on European diving beetles undertaken by Bergsten et al. (2012) found that based on 419 sampled *Agabus bipustulatus* specimens, a sample size of 250 specimens was required to be randomly sampled across its range to achieve 95% haplotype recovery. On the other hand, 70 individuals of the same species was necessary to be sampled in order to recover 95% of COI variation when geographic dispersion between a new sample and the closest previous sample was maximized using resampling simulation.

Not all studies find evidence for greatly broadening the scope of comprehensive specimen sampling. Luo et al. (2015) demonstrate the utility of the central limit theorem (CLT), employing a simple resampling scheme along with the modified Michaelis—Menten saturation model. The CLT states that the distribution of the sample mean tends toward the (standard) normal distribution as the sample size increases. It was found that a minimum sample size of only 20 individuals is needed to provide a reliable estimate of genetic polymorphism at the species level on the basis of observed haplotype numbers. The authors note however that sample sizes should be as large as possible, even though new haplotypes will tend to be observed with lower frequency. Compared to present sample size range of 5–10 specimens per species, a slightly larger minimum sample size range of 11–15 individuals per species was recommended by Yao et al. (2017) for widely distributed coastal and inland aquatic salt‐tolerant plant species of the families Poaceae and Chenopodiaceae across seven different genera, based on results obtained through resampling procedures and nonparametric Mann–Whitney *U* tests.

Though not devoted to estimating sample sizes for mitochondrial genes such as COI, using resampling simulation, Hale, Burg, and Steeves (2012) found that a sample size of 25–30 individuals was sufficient to accurately estimate microsatellite allele frequencies in hypothetical populations of hairy wood ants (*Formica lugubris*), kakis (*Himantopus novaezelandiae*), black‐browed albatrosses (*Thalassarche melanophris*), and red squirrels (*Sciurus vulgaris*). The sampling of 25–30 individuals per species for the assessment of genetic diversity via microsatellite loci was also recommended by Pruett and Winker (2008) in an earlier study of song sparrows (*Melospiza melodia*). A more recent simulation study examining minimum sample sizes for accurate estimation of genetic diversity from a large number of single nucleotide polymorphism (SNP) markers in the terrestrial Amazonian plant *Amphirrhox longifolia* found that sample sizes beyond eight are sufficient for genetic diversity assessment and as few as two individuals are needed in order to obtain good estimates of population differentiation (Nazareno, Bemmels, Dick, & Lohmann, 2017). These studies clearly point to the need for large sample sizes in multilocus population genetic studies for the overall assessment of genetic diversity at the species level.

These examples serve to illustrate the fact that, as is the case for species divergence thresholds, there is no one universal sample size that can accurately recover the majority of intraspecific genetic variation across taxa and it appears likely that varying levels of additional sampling will be required within taxa and across geographic ranges (Lou & Golding, 2012). What seems to be clear is the fact that many previous assessments of sample sizes necessary for DNA barcoding studies have underestimated levels of sampling depth that are actually needed in order to recover much of the genetic variation that exists at the species level. Such a trend seems most attributable to restricted geographic sampling and unclear species boundaries, limited funding for adequate specimen retrieval, and human‐mediated mechanisms such as errors accrued during the amplification/sequencing process.

## 4 CASE STUDY: PHILLIPS ET AL. (2015)

Phillips et al. (2015) wished to estimate *sampling sufficiency* (*θ*)—the sample size at which accuracy is maximized and above which no additional sampling information is likely to be gained. This was applied in the context of haplotype accumulation curves in order to determine the point on the *x*‐axis where curve saturation first becomes evident. If such an estimate exists, it would provide a useful stopping rule for specimen sampling (Phillips et al., 2015). That is, if a lower bound for specimen sample size exists, then it would provide the best estimate of sampling sufficiency for a given species.

### 4.1 Model assumptions

In developing their sampling model, Phillips et al. (2015) made several important assumptions, which together form a baseline “perfect‐world” scenario for further exploration of specimen/haplotype sampling. These are as follows:

- that specimen sampling is carried out randomly and without replacement from an infinitely large, panmictic population with constant size;
- that species haplotypes are both biologically real and unique; and
- that species haplotypes occur with equal frequency.

In the first assumption, the contribution of genetic drift is presumed to be negligible and it is assumed that population structure is absent. Luo et al. (2015) presumed a constant population size, as well as an absence of natural selection, when calculating intraspecific sample sizes for their simulation study. The argument was that a limited number of individuals would be available in species populations undergoing contraction and that coalescence may not be evident. With regard to the second assumption, DNA barcodes are presumed to be of sufficiently high quality such that they are free of both ambiguous and missing nucleotide bases, which can lead to overestimation of observed and total haplotype numbers through creating artificial haplotype variation within species (Athey, 2013; Dasmahapatra, Elias, Hill, Hoffman, & Mallet, 2010; Phillips et al., 2015; Stoeckle & Kerr, 2012; Stoeckle & Thaler, 2014).

Assumptions 1 and 3 were employed by Dixon (2006) in proposing a method to assess the extent of haplotype sampling completeness utilizing a Bayesian statistical framework based on the use of Stirling numbers. It was noted that the probability of all haplotypes being observed for a species becomes less accurate if the assumptions of random sampling and equal haplotype frequencies are not met and that the presence of rare species haplotypes will lead to overestimation of overall sampling completeness. Similarly, Phillips et al. (2015) hypothesized that the presence of rare haplotypes within species will lead to inflation of total sample sizes. Further, as noted by Dixon (2006), evolutionary mechanisms such as isolation by distance, which describes the variation in genetic composition of species populations with increasing geographic distance, will likely cause the true extent of sampling effort to be overestimated. In exploring coalescent simulations, Luo et al. (2015) treated barcode sequences as panmictic. In this way, all specimens can be regarded as being sampled from a single geographic region. Such an assumption is not uncommon within DNA barcoding studies, which are often geographically focused (Collins & Cruickshank, 2013). While Luo et al. (2015) did not consider spatial heterogeneity within their simulation study, it was proposed that stratified sampling, where individuals are repetitively sampled without replacement from a preselected number of strata, can be employed, with the added assumption that gene flow can largely be ignored.

### 4.2 Mathematical details

Phillips et al. (2015) derived a simple Method of Moments (Pearson, 1894) estimator in order to predict adequate specimen sample sizes necessary to uncover the majority of cytochrome *c* oxidase subunit I (COI) DNA barcode haplotype diversity existing within animal species according to the equation

(7) $$N^{*}=\left[\frac{NH^{*}}{H}\right].$$

Above, N* is considered an estimate of *θ*, the true sampling sufficiency, which, under the Frequentist statistical paradigm, is a fixed but unknown parameter. The quantity [*N*/*H*] is the number of specimens represented by each haplotype ([*x*] is the ceiling function applied to a number *x*, evaluated by rounding up to the nearest integer). Since haplotypes are assumed to be sampled with equal frequency from a species population, in a sample of *N* = 100 sequences comprising *H* = 10 distinct haplotypes, it is expected that each haplotype is represented by 10 specimens (Phillips et al., 2015). H* is found using the equation

(8) $$H^{*}=\sum_{i=1}^{H}i=\frac{H(H+1)}{2}$$

where *N* is the number of DNA sequences observed for a given species, *H* is the number of observed haplotypes, and H* is the estimated total number of haplotypes (both observed and unobserved) for a species. The above estimator is similar to estimators of total species richness used widely in ecological settings (e.g., the Chao1 estimator of abundance Chao, 1984). The central idea around the above estimator is that the majority of haplotypes within a species are rare, being represented by only one (singleton) individual. Thus, once such haplotypes have been accounted for in a species sample, few additional unduplicated haplotypes are likely to be observed, since the majority of remaining haplotypes will be dominant (duplicates) in the population (i.e., being represented by two or more specimens); thus, species comprising many singleton haplotypes should be expected to require larger sample sizes in order to capture most of the existing genetic variation for a given species of interest (Phillips et al., 2015; Williams, Huang, Rasmont, & An, 2016).

Phillips et al. (2015) also proposed both absolute and relative “measures of sampling closeness” in order to quantify the extent of specimen and haplotype sampling effort. These quantities are as follows:

- Mean number of haplotypes sampled: *H*
- Mean number of haplotypes not sampled: H*–*H*
- Proportion of haplotypes sampled: $\frac{H}{H^{*}}$
- Proportion of haplotypes not sampled: $\frac{H^{*}-H}{H^{*}}$
- Mean number of individuals not sampled: N* – *N*

**Figure 2:**
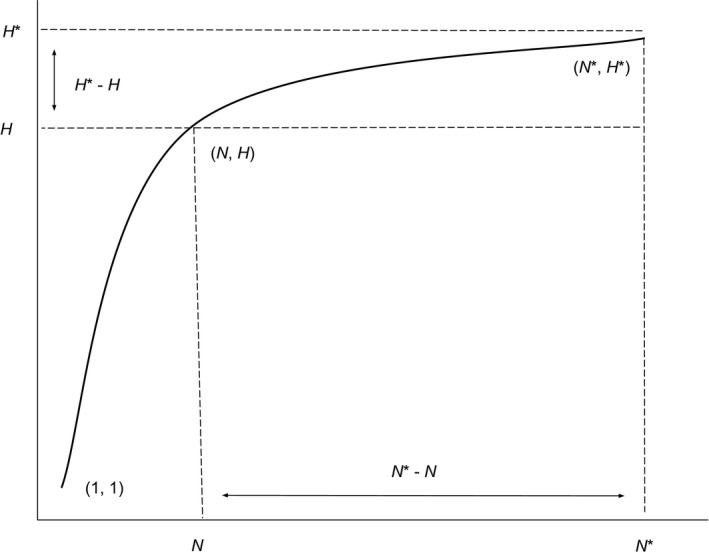
Graphical depiction of Phillips et al.'s (2015) sampling model as described in detail within the main text. The *x*‐axis is meant to depict the number of specimens sampled, whereas the *y*‐axis is meant to convey the cumulative number of unique haplotypes uncovered for every additional individual that is randomly sampled. *N* and *H* refer to specimen and haplotype numbers that are observed for a given species. N* is the total sample size that is needed to capture all H* haplotypes that exist for a species

The above equations, which are central to Phillips et al.'s (2015) sampling model, can be depicted graphically as follows (Figure 2).

Figure 2 resembles the general shape of a saturated haplotype accumulation curve for a hypothetically well‐sampled species. The point labeled (*N*,* H*) on the curve reflects the current level of sampling effort that has been expended for a given species (i.e., as found in BOLD). The goal is to extrapolate the curve to the point (N*, H*) in order to observe the value on the *x*‐axis (i.e., N*) at which leveling off toward an asymptote (on the *y*‐axis) first becomes evident (i.e., at the value of H*). Here, $N^{*}-N$ is the number of additional specimens that must be randomly sampled in order to observe $H^{*}-H$ additional haplotypes for a given species. If *H* is equal to H*, then N* will be equal to *N*, and no further sampling is necessary; otherwise, if *H* is less than H*, then N* will be greater than *N*, and additional sampling will be required. The curve in Figure 2 passes through the point (1, 1), which is due to the fact that the sampling of a single individual of a given species corresponds to observing one unique haplotype for that species.

### 4.3 Application to ray‐finned fishes

Phillips et al. (2015) investigated levels of existing COI haplotype variation in 18 species of ray‐finned fishes (Chordata: Actinopterygii) represented by a minimum of 60 individuals in accordance with Grewe et al. (1993). Results showed that 147–5,379 specimens likely must be randomly sampled to uncover all predicted haplotype diversity in the selected species (between 3 and 528 total haplotypes) (Phillips et al., 2015). Sample size estimates obtained by Phillips et al. (2015) are comparable in magnitude to those of Zhang et al. (2010), but not in the case of Luo et al. (2015), which are closer to practical sample sizes for DNA barcoding. Further, haplotype accumulation curves displayed evidence of reaching an asymptote for only 3/18 examined species: Chinook salmon (*Oncorhynchus tshawytscha*), Rockfish (*Sebastes* sp.), and Siamese fighting fish (*Betta splendens*) based on significance testing of curve slopes with a one‐sided *t* test using the last 10 points on the end of accumulation curves (Phillips et al., 2015). Of note is the haplotype accumulation curve for Chinook salmon, which appeared to show premature saturation despite only 12 out of an estimated total of 78 haplotypes being found for the species. At the time of publication of Phillips et al.'s (2015) study, *Sebastes* sp. was linked to a single BIN. The BIN system is inherently dynamic: As more sequences are added within BOLD, specimens assigned to a single BIN may be allocated to multiple BINs or multiple existing BINs may be coalesced into a single BIN. This is especially the case as species boundaries become clearer or taxonomic revisions are made. As an example, the genus *Sebastes* is a highly speciose group, thought to have undergone an adaptive radiation as recently as 8–9 million years ago (Steinke, Zemlak, Boutillier, & Hebert, 2009). This fact could explain the low haplotype diversity observed for this species (two haplotypes across 98 individuals). Such findings may be due to the underlying assumptions of the model, which are likely to be over‐simplistic, particularly that of equality of intraspecific haplotype frequencies. Further, the proposed estimator for the calculation of total haplotype diversity (H*) (Equation (7)) may be a gross overestimate. Despite not being realistic for populations of real species, the reason for adopting a uniform distribution of haplotypes was due to mathematical convenience, in order to make calculations of sample size as simple and as straightforward as possible. This is commonly done in practice, since determining the true distribution of species haplotypes is likely strongly dependent on species under study. Thus, values of N* are likely overestimates of the true number of specimens that must be randomly sampled in order to observe most haplotype variation that exists for a species (Phillips et al., 2015). Phillips et al. (2015) argue that the use of a limited number of points in the calculation of curve slopes may not be adequate; the authors argue that a fixed proportion of curve points should instead be used. Further, through successively targeting the last 20%–15%, 15%–10%, and the last 10% of species haplotype accumulation curves, in order to observe a statistically significant change in slope values, the precise point of saturation can be localized (Phillips et al., 2015).

Determining the precise point corresponding to haplotype accumulation curves reaching an asymptote (i.e., having a slope near zero) is difficult. One way this can be accomplished is through employing numerical techniques, specifically iteration. Such methods work by repeatedly recycling computed values into an algorithm; that is, current values are used as starting values to the next iteration until convergence to a solution is achieved. One way this can be realized is through iterating Equation (7) along with the equations for the “measures of sampling closeness” proposed by Phillips et al. (2015). This seems to be the most logical way forward in better ascertaining at what level specimen sampling is deemed sufficient and thus when further collection of specimens should be ceased.

## 5 FUTURE PROSPECTS

The present review explores the issue of sampling in DNA barcoding from the perspective of computational and statistical methodologies. Key sample size studies in the barcoding literature were examined in detail. A lack of consensus exists in the most appropriate number of specimens that must be targeted in order to uncover the majority of haplotype diversity that exists at the species level for a variety of taxa. This question is similar to the problem of calculating species divergence thresholds for taxon delimitation and is strongly dependent on species abundances, life histories, and geographic coverage. To date, few studies exploring sample sizes for DNA barcoding have been conducted. Existing studies (Phillips et al., 2015; Zhang et al., 2010) appear to point to the comprehensive sampling of hundreds to thousands of specimens in order to capture a wide range of standing genetic variation for a given species based on asymptotic behavior of haplotype accumulation curves.

In order to thoroughly examine the issue of determining specimen sample sizes that are necessary for full assessment of COI DNA barcode haplotype sampling completeness within animal species, relaxation of assumptions inherent in Phillips et al.'s (2015) sampling model is necessary. Specifically, subsequent approaches should investigate the following:

1. relaxing the assumption of uniformity of species haplotype frequencies;
2. loosening the assumption of panmixia within species; and
3. testing both above assumptions in tandem.

The incorporation of population structure into models of haplotype sampling is not straightforward, as sampling strategies for DNA barcoding are quite variable and highly dependent on the taxa under study. Thus, this necessitates the introduction of a more spatially explicit systematic sampling (e.g., phylogeographic) of species genetic variation across distinct taxon boundaries and along phenotypic gradients (i.e., clines). The view of DNA barcoding metaphorically as a “molecular transect,” along which a wide range of intraspecific haplotype diversity can be uncovered, is fitting. Within‐species genetic variation has been limited to over‐representation of deep sampling of a single or a few populations. If the ultimate goal is to account for levels of standing genetic variation with species, then constraining taxon sampling to narrow geographic regions is not ideal, as this can be considered a form of pseudo‐replication. This seems to be an issue of nestedness in sampling and while some depth of sampling within a population is certainly warranted, it cannot be conflated with depth of sampling across populations within a species. In addition, future research should aim to answer the question: Is there an optimal threshold for specimen sampling above which no new DNA barcode haplotype variation is likely to be observed for a species? While it should be possible to find this limit for already well‐sampled taxa based on trends seen in haplotype accumulation curves, the use of haplotype accumulation curves to estimate sample sizes that are required for full assessment of COI DNA barcode haplotype sampling completeness has only been tested in one previous study (Zhang et al., 2010). Phillips et al. (2015) expanded on previous studies through proposing a simple and easily implemented method to estimate specimen sample sizes for a number of ray‐finned fish species, which are among the most densely sampled to date within BOLD. Sample size optimization for the identification of animal species across wide‐ranging geographic scales is key since intraspecific variation within DNA barcodes is not easy to measure, and obtaining large numbers of barcodes that reflect a wide range of intraspecific genetic divergence is sometimes challenging (Bertolazzi, Felici, & Weitschek, 2009). In addition to being able to report likely required specimen sample sizes necessary to achieve saturation in species haplotype curves, it would be ideal if DNA barcoding studies could also provide a global measure of geographic dispersion in order to reliably test for cases of isolation by distance within species. Unfortunately, no such measure yet exists in this regard, making these kinds of analyses problematic. While model estimates may not be practical, having such a framework at hand that easily allows for the calculation of lower bounds for sample size offers researchers a glimpse into the most appropriate taxon sample sizes to target, and potentially where those taxa should be sampled. More crucially, the present simulation proposed herein can be employed in order to best determine the proper allocation of sampling effort, time, and resources (Hortal & Lobo, 2005). Such work finds application in studies of metabarcoding (Wares & Pappalardo, 2015) as well as more broadly to global climate change (Pfenninger, Bálint, & Pauls, 2012).

The development of a computational simulation of haplotype accumulation curves, a tool that can greatly aid biodiversity scientists in targeting species that will benefit from increased sampling effort, can be employed in order to build and grow BOLD with statistically defensible taxon records, which ultimately will allow more reliable specimen identification. This work is crucial because many taxon records currently in BOLD are known from only single specimens. Further, such a simulation algorithm could aid in species discovery through providing more reliable estimates of intraspecific sample sizes used in the calculation of the barcode gap. Through developing statistically relevant sample size estimation tools that capture geographic and genetic variation within and between species, researchers will be able to improve sampling design strategies, which will lead to a better understanding (and improved database) of intra‐ and interspecies genetic variation. As such, new methodologies will fill this void and contribute to the growing literature on sample size estimation for DNA barcoding as well as be implemented as another tool to add to the biodiversity toolbox.

## CONFLICT OF INTEREST

None declared.

## AUTHOR CONTRIBUTIONS

JDP conducted the literature review and wrote the manuscript. DJG acted as an advisor in statistics. RHH acted as an advisor in DNA barcoding. All authors contributed to the revision of this manuscript and approved the final version.

## Data Availability

DNA barcodes used in generating the haplotype network can be found on FigShare (https://doi.org/10.6084/m9.figshare.6281543).
